# Diagnostic Utility of Red Flags for Detecting Spinal Malignancies in Patients with Low Back Pain: A Scoping Review

**DOI:** 10.3390/jcm14207174

**Published:** 2025-10-11

**Authors:** Gianluca Notarangelo, Michele Margelli, Giuseppe Giovannico, Francesco Bruno, Claudia Milella, Daniel Feller, James Dunning, Lorenzo Storari, Firas Mourad, Filippo Maselli

**Affiliations:** 1 Department of Medicine and Health Science “Vincenzo Tiberio”, University of Molise, c/o Cardarelli Hospital, C/da Tappino, 86100 Campobasso, Italy; g.notarangelo1@studenti.unimol.it (G.N.); giuseppe.giovannico@unimol.it (G.G.); francescobrunobb@gmail.com (F.B.); 2 Department of Human Neurosciences, Sapienza University of Rome, 00185 Rome, Italy; mrgmgl@unife.it (M.M.); c.milella94@gmail.com (C.M.); lorenzo.storari93@gmail.com (L.S.); 3 Provincial Agency for Health of the Autonomous Province of Trento, 38100 Trento, Italy; d.feller@erasmusmc.nl; 4 Department of General Practice, Erasmus MC, University Medical Centre, 3015 CA Rotterdam, The Netherlands; 5 American Academy of Manipulative Therapy Fellowship in Orthopaedic Manual Physical Therapy, Montgomery, AL 36104, USA; drjamesdunning@googlemail.com; 6 Montgomery Osteopractic Physical Therapy & Acupuncture Clinic, Montgomery, AL 36104, USA; 7 Department of Health, LUNEX University of Applied Sciences, Differdange, Luxembourg L-4671, Luxembourg; firas.mourad@me.com; 8 Luxembourg Health & Sport Sciences Research Institute A.s.b.l., Differdange, Luxembourg L-4671, Luxembourg; 9 Facoltà Dipartimentale di Medicina e Chirurgia, Università Campus Bio-Medico di Roma, 00128 Roma, Italy; 10 Sovrintendenza Sanitaria Regionale Puglia INAIL, 70126 Bari, Italy

**Keywords:** low back pain, red flags, cancer, scoping review, diagnosis, clinical reasoning, malignancy

## Abstract

**Introduction**: While low back pain (LBP) is most often associated with musculoskeletal issues, in a minority of cases, it can be caused by serious underlying conditions such as cancer. Recognizing malignancy early remains a major clinical challenge, as the warning signs, known as red flags (RFs), are often vague and inconsistent. **Methods**: A comprehensive search of six databases (PubMed, Scopus, Google Scholar, Web of Science, Cochrane Library, and SciELO) and grey literature was conducted for studies published from January 1999 to March 2025. Eligible sources included studies describing adult patients with cancer presenting with LBP. Study selection and data extraction were independently performed by two reviewers. **Results**: We included 70 studies, most of which were case-based, along with reviews and observational research. In these studies, cancer prevalence among patients with LBP ranged from 0.1% to 1.6%, with metastatic disease being the most common finding. A prior history of cancer emerged as the most reliable red flag (specificity up to 0.99), while other signs and symptoms were less consistent. Notably, combining multiple RFs, such as a history of cancer and unexplained weight loss, significantly improved the diagnostic accuracy (LR+ = 10.25 in one study). **Conclusions**: While current evidence is limited and largely based on case-based studies, some RFs, particularly a history of cancer, show greater diagnostic value. In patients with LBP associated with underlying malignancy, RFs seem to be more useful for ruling in rather than ruling out (i.e., screening) serious pathologies. Most RFs have poor standalone accuracy; however, considering combinations of RFs within the broader clinical context may improve early detection of spinal malignancy in patients with LBP.

## 1. Background

Low back pain (LBP) refers to pain or discomfort occurring between the lower ribs and the gluteal folds with or without leg pain [1,2,3,4,5]. When no specific cause, such as infection, tumor, fracture, peripheral arterial disease, or nerve involvement, is identified, it is classified as non-specific LBP [1,2,3,4,5]. In recent years, LBP has been recognized as the most common musculoskeletal disorder and the leading cause of disability worldwide [6,7]. In the primary care setting, between 1% and 5% of all patients who present with LBP have a serious spinal pathology [8]. Among these serious conditions, cancer is the second most frequently reported cause mimicking LBP, with prevalence reported up to 2.1% of cases, while spinal fractures are the most frequent, with prevalence up to 7.2% [9,10]. According to the World Health Organization (WHO), cancer remains the second leading cause of death globally [11]. These serious conditions are often associated with pathognomonic signs/symptoms or red flags (RFs), as they serve as clinical alerts to prompt referral to a medical physician for further investigation [12]. Physiotherapists must be able to recognize sinister presentations that may require further investigation or urgent referral [13].

Delayed recognition of spinal malignancies may result in misdiagnosis and rapid disease progression without the appropriate intervention; therefore, for the primary purpose of identifying relevant RFs, a thorough history and physical examination is essential when encountering patients with LBP [14]. Notably, when only low- or intermediate-risk RFs are present, a watchful waiting approach may be appropriate to monitor progress to avoid a delayed or missed diagnosis of a serious disease [15]. When RFs with high diagnostic value are identified, a timely investigation and referral to a medical physician or spinal specialist is warranted. Screening for these indicators must be a priority and core responsibility for physiotherapists who are managing patients with spinal pain [16]. However, screening tools have limitations and may not be sufficient to definitively rule out serious underlying conditions [17].

This scoping review aimed to map and synthesize the literature; furthermore, it aimed to describe the relevant RFs associated with cancer-related cases of LBP.

## 2. Materials and Methods

This scoping review has been conducted in accordance with the methodology from the Joanna Briggs Institute (JBI) [18]. In addition, the “Preferred Reporting Items for Systematic Reviews and Meta Analyses extension for Scoping Reviews (PRISMA ScR)” checklist [19] has been used for reporting the findings.

### 2.1. Research Question

The following research question was formulated:

“What is known from the current literature on RFs for cancer in patients presenting with LBP?”

### 2.2. Protocol and Registration

The scoping review protocol was published on 1 September 2024 and is available online at: *medRxiv* 2024.09.01.24311072 [20].

### 2.3. Inclusion Criteria

Studies were included if they fulfilled the following criteria regarding population, concept, and study context (PCC criteria):

Population: patients of any age and gender.

Concept: studies that have explored and reported RFs associated with LBP caused by any cancer pathology. We defined RFs as clinical findings—i.e., signs and/or symptoms from the history and/or physical examination—that may indicate serious conditions (e.g., cancer) and are used to support early detection and appropriate referral [12].

Context: studies conducted in any setting and geographic context, published in English or Italian, from January 1999 to March 2025.

### 2.4. Exclusion Criteria

Studies that did not meet the above PCC criteria were excluded.

### 2.5. Search Strategy

The literature search was conducted on the following databases until 15 March 2025: MEDLINE (via PubMed), Scopus, Google Scholar, Web of Science, Cochrane Library and SciELO.

The complete search strategy for all databases can be found in the Appendix A.

### 2.6. Selection of Studies

Search results were imported into EndNote V.X9 (Clarivate Analytics, PA, USA), and duplicates were removed. Two independent authors (GN, FB) screened records at two levels (title/abstract and full-text) using Rayyan [21]; disagreements were resolved by a third author (FM). Study design classification followed authors’ descriptions and methodology. Systematic reviews were identified by structured search strategies, eligibility criteria, and formal quality or bias assessment, in line with PRISMA [22]. Scoping reviews were classified according to exploratory aims and broad inclusion criteria (PRISMA-ScR [19]), while narrative reviews were defined as overviews without reproducible methodology [23]. Additional articles were categorized as guideline review or editorial.

### 2.7. Data Extraction

Data extraction followed the PCC model using a standardized Excel sheet. Recorded information included study details (author, year, country, design), population characteristics, and clinical data (case description, diagnosis, out-comes). Diagnostic accuracy measures were noted only when explicitly reported in the original studies. Two independent authors (CM, GG) extracted data, with disagreements resolved by a third author (FM). Full data are available in Appendix B.

### 2.8. Data Synthesis

Data were summarized and collected as a descriptive analysis. A mapping of the data was carried out that showed the distribution of the studies by publication period, study design and topics. A thematic synthesis of cancer-specific RFs was performed in patients presenting with LBP. Additional descriptive analyses of subgroups (e.g., gender, pathology, etc.) were reported. In line with the Joanna Briggs Institute (JBI) methodology for scoping reviews [18], no formal assessment of methodological quality or risk of bias was performed, as the aim was to map and synthesize the available evidence rather than appraise the study validity.

## 3. Results

### 3.1. Characteristics of the Included Studies

**Figure 1:**
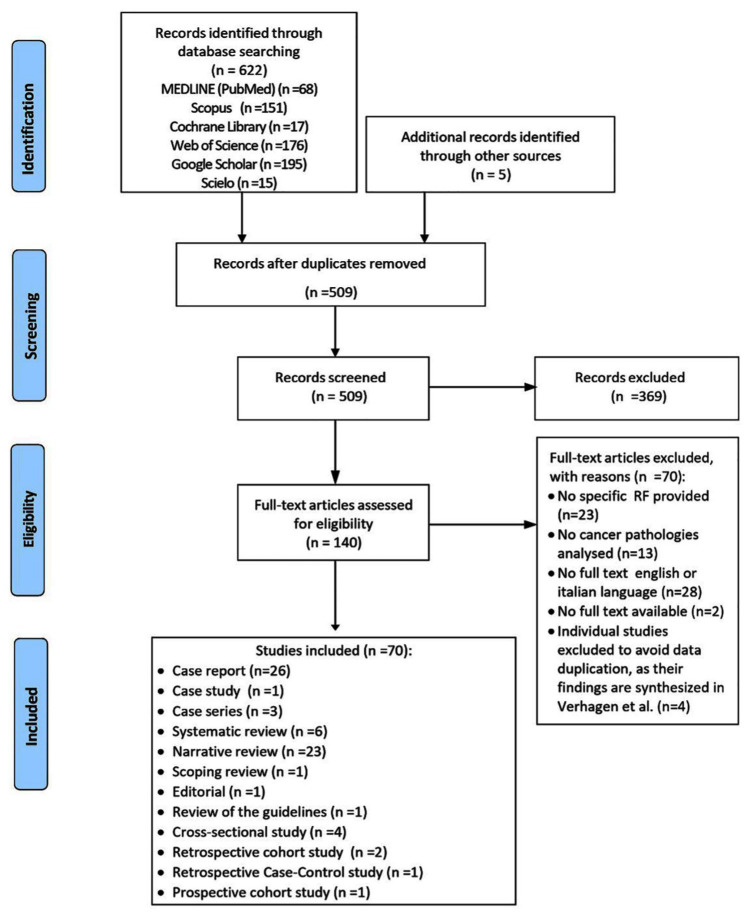
PRISMA Flowchart.

The PRISMA-ScR flowchart (Figure 1) illustrates the study selection process. From 627 records initially identified (including grey literature), 118 duplicates were removed. Following title/abstract and full-text screening, 70 studies fulfilled the eligibility criteria and were included in this scoping review. Four studies (three case reports [24,25,26] and one case study [27]) were excluded at this stage as their data were already synthesized in Verhagen et al.’s systematic review [28]. Detailed reasons for exclusion are provided in Appendix C. The selected studies include 27 case reports [29,30,31,32,33,34,35,36,37,38,39,40,41,42,43,44,45,46,47,48,49,50,51,52,53,54,55], 3 case series [56,57,58], 6 systematic reviews [10,17,28,59,60,61], 1 scoping review [62], 23 narrative reviews [14,15,16,17,18,19,20,21,22,23,24,25,26,27,28,29,30,31,32,33,34,35,36,37,38,39,40,41,42,43,44,45,46,47,48,49,50,51,52,53,54,55,56,57,58,59,60,61,62,63,64,65,66,67,68,69,70,71,72,73,74,75,76,77,78,79,80,81,82,83] 1 review of the guidelines [84], 1 editorial [85], 4 cross-sectional studies [86,87,88,89], 2 retrospective cohort studies [90,91], 1 prospective cohort study [92], and 1 case–control study [93].

The studies were conducted primarily in the United States (39%), Italy (10%), and Brazil (8%). Thirty of the included studies were conducted from 1999 to 2014, while the remainder were conducted in the last decade (Table 1).

### 3.2. Patient Assessment and Clinical Findings

#### 3.2.1. Case Reports, Case Series, Case Studies

Several comorbidities were reported, including cardiac or vascular diseases (i.e., hypertension and cerebrovascular disorders [31,36,37,56]), type 2 diabetes [31,37,45] and gastrointestinal diseases [31,39,56]. In addition, hip dysplasia [55], osteoporosis [49,55], obesity [34,37], and gynecological diseases [32,42] were reported. Neurological disorders (cerebrovascular disease) [31], kidney disease (presence of stones) [36], hypothyroidism [36], and a rheumatic disease (scleroderma with Raynaud’s phenomenon) [55] were also reported. Notably, 16 patients reported no comorbidities.

Considering laboratory tests, blood work was requested in 17 patients and biopsies were requested for 20 patients. The most requested imaging examinations were magnetic resonance imaging (MRI) and X-rays in 23 cases, and computed tomography (CT) in 18 cases. There were 23 cases of direct access to hospitals, 3 cases of direct access to a physiotherapist, and 6 cases of direct access to a chiropractor. Two patients reported a history of smoking or alcohol use [34,52].

In patients with LBP associated with underlying serious pathologies, Table 1 demonstrates that the presence of sinister signs and symptoms has a heterogeneous distribution. Lumbar tenderness was reported in 7 cases [29,31,34,35,43,55,56] and the presence of radiating pain was reported in 14 cases [31,32,33,35,36,38,41,43,46,47,50,53,56] (2 cases in the same study [56]). Neurological signs (such as reduced sensitivity, loss of sphincter control, gait impairment, and changes in osteotendinous reflexes) were reported in 22 cases [29,30,31,32,33,35,36,38,39,40,41,46,47,49,50,51,52,53,54,55,56]; muscle weakness was reported in 9 cases [31,33,38,46,53,54,55,56]. Both findings included two patients described in the same case study [56].

Patient characteristics (e.g., gender, age, setting, comorbidities, clinical signs, imaging findings, and outcomes) from the studies included in this review have been summarized in Table 1. Additionally, Table 1 summarizes the 30 case-based studies (26 case reports, 1 case study, and 3 case series) included in the analysis; notably, there are 32 patients in total as two studies described more than one case [56,57] and in one case series [58] only one patient presented with LBP.

#### 3.2.2. Observational Studies

In addition to case-based studies, eight observational studies offered further insight into patient assessment and clinical presentation in broader populations.

Chu et al. conducted a retrospective analysis of 20 patients with cancer-specific LBP in chiropractic clinics [88]. All patients underwent MRI, and 18 reported symptoms radiating to the lower limbs; further, the pain onset was classified as acute (less than 6 weeks) in 10 cases and chronic (more than 12 weeks) in 18 cases. Reported comorbidities included a history of cancer and hypertension (three cases each), hyperlipidaemia, cardiovascular disease, and diabetes. No comorbidities were reported in 12 of the patients. Heterogeneous RFs were found, including progressive symptoms in three patients, while night pain, pain at rest, bilateral radiculopathy, and increased urinary frequency were each reported in one patient [88].

Reito et al. evaluated 900 emergency department visits with acute or subacute atraumatic low back pain with or without radicular symptoms and found only 3.7% of cases were due to a specific spinal pathology. Nevertheless, the low incidence of spinal pathologies reflects the limited predictive accuracy of RF findings in this setting [86].

In patients undergoing surgery for spinal metastases, Van Tol et al. reported that back pain was the most common presenting symptom and was often accompanied by neurological deficits; however, the referral documentation frequently failed to record these specific clinical findings, even when they were present at the time of patient assessment [89]. Likewise, Henschke’s prospective cohort of 1172 primary care patients with acute LBP reported serious spinal pathology, such as cancer, in patients over the age of 50, with a history of malignancy, or with unexplained weight loss [92].

Premkumar et al. observed that malignancy-related LBP is often clinically indistinguishable from benign presentations, with RFs like prior cancer history or advanced age not always accompanied by specific alarming signs [90]. Collectively, these studies confirm that cancer-related LBP frequently mimics non-specific presentations, with RFs and comorbidities emerging only after more in-depth assessment [90,92].

### 3.3. Cancer-Specific RFs

#### 3.3.1. Case Reports, Case Series, Case Studies

The most frequently reported RF was “neurological signs” cited in 22 cases. Other relevant RFs were: “history of cancer”, “family history of cancer”, “failure of conservative treatment”, “age over 50”, “progressive worsening pain”, “night pain”, pain duration over 3 months, and “severe and continuous pain”.

Table 2 below summarizes the RFs identified in the included case reports, case studies, and case series. For each case, RFs were either explicitly reported in the article or inferred from the information provided in the onset, clinical presentation, and physical examination. All terms were standardized to predefined terminology. In addition to the column indicating whether each RF was reported by the authors because of clinical findings during the history and physical examination, a further column specifies the number of studies in which the RF was not reported.

#### 3.3.2. Observational Studies

Several primary studies investigated the diagnostic performance of RFs in patients with LBP, with considerable variability in sensitivity (Se), specificity (Sp), and likelihood ratios (LR) depending on the individual feature and the study setting.

Demographic-related RFs such as “age > 50” were among the most frequently examined. Notably, in the prospective cohort by Henschke et al. [92], this RF (i.e., “age > 50”) showed a specificity of 0.660 (95% CI 0.630–0.690), whereas, in Premkumar et al. [90], it dropped to 0.326 with an LR+ of only 1.060, suggesting poor diagnostic accuracy when considered alone.

Raising the threshold to “age > 70” increased the specificity to 0.795 (0.950 in Henschke’s study [92]); however, the sensitivity dropped to 0.220 in comparison to the previous “age > 50” in the same study. Demographic-related RFs can be viewed in Table 3.

Medical history-related RFs, particularly a “history of cancer,” consistently demonstrated the strongest diagnostic accuracy across studies. In Premkumar et al. [90], a specificity of 0.956 and an LR+ of 7.250 were reported, with Tsiang et al. [93] reporting similar values for diagnostic accuracy (Sp 0.778; Se 0.917). Although Henschke et al. [92] found zero sensitivity for this indicator, the specificity remained high at 0.960, supporting its strong role of ruling in a diagnosis when a history of cancer is confirmed. Likewise, “unexplained weight loss” showed poor sensitivity across all studies (0.082 in Premkumar [90], 0.000 in Henschke [92]), but excellent specificity (0.956 in Premkumar [90] and 1.000 in Henschke [92]), reinforcing its limited value in ruling out malignancy, but strong utility in reinforcing diagnostic suspicion when present. Clinical symptoms such as night pain and pain at rest were also explored, though with less favorable results. Tsiang et al. [93] found that night pain had a sensitivity of 0.542 and specificity of 0.496 indicating lower diagnostic utility. Notably, pain at rest fared even worse with a sensitivity of 0.250. Among neurological or systemic signs, urinary retention possessed high specificity (0.958) but low sensitivity (0.042).

Overall, and in patients with LBP associated with underlying malignancy, RFs seem to be more useful for ruling in rather than ruling out (i.e., screening) serious pathologies. Medical history-related RFs are reported in Table 4.

The physical examination-related RFs were generally consistent with this trend. In the cohort by Henschke et al. [92], altered sensation had a high specificity (0.980), but close to zero sensitivity. More specifically, this impairment does not effectively exclude malignancy; however, the presence of such should heighten concern. No additional relevant data on physical examination-related RFs were reported in the other included primary studies. Physical examination-related RFs can be consulted in Table 5.

Importantly, combining multiple RFs considerably improved diagnostic performance. Premkumar et al. [90] demonstrated that the combination of “history of cancer” with “unexplained weight loss” gave an LR+ of 10.250, significantly increasing the post-test probability of spinal malignancy. The diagnostic accuracy of combined RFs is reported in Table 6.

#### 3.3.3. Review Studies

Additional combinations of RFs have been described in secondary literature, although these findings were not extracted from primary studies. Importantly, the primary studies referenced within these secondary sources are not included among the studies selected for the present scoping review; therefore, their reported diagnostic values should be interpreted with caution and considered as indirect evidence rather than as part of the primary data synthesis.

These proposed clusters may serve as reference points for clinical reasoning, but their diagnostic value should be interpreted with caution, given the absence of original patient-level data. For instance, Delladio et al. [82] reported a combination including age > 50 years, history of cancer, unexplained weight loss, and failure to improve after one month, yielding a sensitivity of 1.000, specificity of 0.600, LR+ of 2.400, and LR− of 0.060. Similarly, the systematic review by Henschke et al. [60] described the same cluster with a sensitivity of 1.000, though without a corresponding specificity estimate. In line with these observations, Finucane et al. [63] similarly noted that most RFs beyond ‘history of cancer’ remain poorly validated and often present too late to aid early detection. The diagnostic accuracy of combined RFs is reported in Table 7.

### 3.4. Differential Diagnosis

#### 3.4.1. Case-Based Studies

The most frequently reported diagnosis was “secondary metastatic lesions” (17 cases), as described in 16 different sources [29,30,31,34,35,36,39,40,41,44,45,47,48,52,55,56]; notably, one of these studies reported two distinct metastatic cases [56], accounting for the total of 17. Primary cancers were the second most cited diagnosis, with 15 cases identified across the literature. The different cancer-related diagnoses included Ewing’s sarcoma [32,41,51], osteoid osteoma [57], lumbar schwannoma [33,43], and primary bone liposarcoma [38]. Other rare conditions reported in single cases were cavernous angioma [46], giant cell cancer [54], spinal neurofibroma [53], mastocytosis [49], renal angiomyolipoma [50], and leukemia [44]. The metastatic lesions were highly heterogeneous and originated from various primary sites, including the stomach, lung, colon, prostate, breast, pancreas, urothelium, appendix, pelvis, penis, and spine.

#### 3.4.2. Observational Studies

Among the observational studies included, several provided data relevant to the differential diagnosis of malignancy-related LBP. In the study by Premkumar et al. [90], conducted on a cohort of 2505 patients, 35 were diagnosed with serious pathology. More specifically, metastatic disease was found in 18 cases (51%), while primary cancers such as lymphoma (2 cases), multiple myeloma (2 cases), and Ewing sarcoma (1 case) were much less common. The remaining 12 cases included infections, fractures, and inflammatory pathologies.

Henschke et al. [92], in a prospective cohort of 1172 patients in a primary care setting, reported 11 cases of serious spinal pathology. Notably, only two cases were cancers: one individual with prostate cancer and one individual with multiple myeloma. The study confirmed that cancer is rare in this context (around 0.17%), which highlights how difficult it can be to spot serious conditions during the initial evaluation.

Tsiang et al. [93], in a retrospective analysis of 7221 self-reported LBP cases, identified 20 patients with confirmed cancer-related diagnoses. Of these, 18 were secondary metastatic lesions (90%), while only two (10%) were due to primary cancers. Although the exact distribution was not detailed, the most frequently involved cancers were prostate, breast, lung, and gastrointestinal.

Reito et al. [86] investigated 790 patients attending the emergency department and identified 79 cases (10%) of specific spinal pathologies. Among these, 13 patients (1.6%) were diagnosed with cancer, including six with metastatic disease, four with multiple myeloma, and two with lymphoma. Other serious conditions included vertebral fractures (n = 19) and spinal infections (n = 6), illustrating the broad spectrum of differential diagnoses in acute atraumatic low back pain.

Chu et al. [88] conducted a retrospective study within chiropractic clinics, analyzing 20 patients with LBP ultimately diagnosed with malignancy. Notably, 18 of these patients (90%) had metastatic lesions, while only 2 (10%) had primary cancers. Furthermore, most lesions originated from prostate, breast, lung, and urogenital cancers. This study also revealed that many patients first sought help from chiropractors, suggesting that non-medical settings can play an important role in raising early suspicion and prompting referral for serious conditions.

Van Tol et al. [89] retrospectively analyzed 389 patients treated surgically for symptomatic spinal metastases. The distribution of primary cancer sources was as follows: urogenital cancers (21.9%), hematological malignancies (19.8%), breast cancer (18.8%), lung cancer (14.9%), gastrointestinal cancer (6.4%), gynecological cancer (1.5%), and other or unspecified causes (16.7%). This study emphasized the diagnostic complexity and delayed diagnoses frequently observed in cancer-related LBP.

Finally, Erausquin et al. [91] presented a case series of Naka grade III lumbar epidural lipomatosis, of which only one case reported LBP as a primary symptom. The diagnosis was a benign epidural lesion, suggesting how important it may be to also consider rare, non-cancer causes when evaluating patients with LBP.

## 4. Discussion

This scoping review aimed to map the existing literature about RFs described in patients with cancer-related LBP. Although the studies varied considerably in their methodological design and level of detail in reporting, formal quality appraisal was not undertaken, in line with current JBI guidance for scoping reviews [18]. Most of the available evidence comes from case-based studies; nevertheless, while these studies are useful for illustrating rare or atypical cases, they are observational in nature and do not provide experimental data to support broad conclusions. Among the 32 patients described in the case-based literature, secondary spinal malignancies represented the most frequent diagnosis, often presenting with nonspecific symptoms such as persistent pain, neurological deficits, or insidious onset. The variety of comorbidities and patient ages highlights how complex these cases can be and how much their presentation can differ from one another. The frequent use of advanced imaging techniques and biopsies indicates that initial presentations were often nonspecific, making early clinical suspicion of malignancy unlikely and sometimes leading to diagnostic delay. The observational studies included in this review collectively offer insight into how cancer is diagnosed in patients with LBP and the types of cancers most frequently identified. Across these studies, metastatic disease emerged as the predominant finding, while primary cancers such as prostate, breast, lung, gastrointestinal, hematologic, and urogenital malignancies were less common [86,87,88,89,90,91,92,93]. The study by Chu [88], which examined both individual case presentations and a broader retrospective sample (n = 7221), confirmed the low overall prevalence of cancer in LBP (0.1–1.6%), but also emphasized that metastatic disease was by far the most common serious diagnosis when cancer was present. In van Tol’s retrospective analysis of surgically treated patients with spinal metastases [89], breast, lung, urogenital, and hematologic cancers emerged as the most frequent primary sites. These findings align with what was observed in the case-based literature and further support the idea that spinal metastases often develop from common types of systemic cancer [29,30,31,35,36]. Similarly, Reito et al. [86] reported hematologic and lung cancers among patients with acute LBP in the emergency setting, reinforcing the importance of considering these etiologies in differential diagnosis.

Most of the systematic, scoping, and narrative reviews identified were relevant to our research question and offered useful context, but they were excluded from the quantitative analysis. Their quality was not formally assessed, as this lies outside the domain of a scoping review. For example, Maselli et al. [17], Henschke et al. [60], and Verhagen et al. [28] discussed approaches to evaluating RFs in suspected serious pathology, including cancer; however, for methodological reasons, their findings were cited only as background rather than included in the statistical synthesis. The limited strength of evidence, as well as conflicting diagnostic accuracy values, were repeatedly highlighted by the authors themselves, justifying a cautious interpretation of the diagnostic utility of RFs.

A main outcome of this scoping review is that most of the evidence derives from case reports or small observational studies. Even reviews in this field rely on very few primary sources, underscoring the scarcity of robust data and thus limiting the strength of epidemiological or clinical inferences. Within this context, some authors proposed specific combinations of RFs, referred to as clusters, as more diagnostically valuable than isolated signs. These clusters, discussed by Henschke [60], Verhagen [28], and Delladio [82], were associated with higher sensitivity and/or specificity values and appear particularly relevant in guiding early referral or diagnostic imaging. In line with these observations, “history of cancer” consistently emerged as the most reliable individual RF [90,92,93], while most others showed limited accuracy when assessed in isolation. Similar conclusions were drawn by Finucane et al. [63], who emphasized that aside from a previous history of cancer, most RFs lack sufficient validation and may present too late to be useful for early detection. This supports the importance of combining RFs with clinical judgment to strengthen diagnostic reasoning. Although the quality of the supporting evidence is only moderate to low, the fact that these combinations are repeatedly mentioned across different sources suggests they may be worth exploring further in clinical research.

Considering these findings, the existing literature on cancer presenting with LBP appears fragmented and often limited to anecdotal or retrospective data. The lack of prospective studies and the predominance of case-based literature limit the development of strong epidemiological conclusions, highlighting the need for better-designed research with larger samples to improve understanding of the diagnostic process. Similar observations have also been made in studies on neck pain, where single RFs showed limited reliability and most serious cases were due to metastatic disease. This suggests that the difficulties in recognizing cancer are not confined to the lumbar spine but can be seen across different spinal regions [94]. A final summary table (Table 8) presents the diagnostic performance of selected individual RFs together with clinical clusters identified across Premkumar’s primary study [90]. For comparative purposes, two additional clusters derived from two reviews are included, although these are secondary data and were not used in the main analysis [60,82].

### 4.1. Implications for Clinical Practice

This scoping review offers valuable insights into the nature and limitations of the current evidence surrounding the diagnostic process for identifying cancer in patients presenting with low back pain. The predominance of case-based studies highlights the lack of high-level research and reinforces the need for clinical caution when interpreting RFs. Although several systematic reviews have attempted to define the diagnostic utility of the RFs, they often rely on limited primary data, and the findings appear inconsistent. Clinicians should avoid over-reliance on individual RFs; more specifically, clinicians should adopt a reasoned clinical approach that considers combinations of risk factors and the broader clinical context. Overall, and in patients with LBP associated with underlying malignancy, RFs seem to be more useful for ruling in rather than ruling out (i.e., screening) serious pathologies. Nevertheless, the poor specificity of many RFs underscores the risk of both under- and over-diagnosis. These findings support the development of more refined clinical tools and structured decision-making frameworks to enhance cancer screening in patients with LBP, particularly in direct access contexts.

### 4.2. Research Implications

More robust evidence is needed to understand how individual RFs, and especially their combinations, can help in the early detection of cancer in patients presenting with LBP. At present, much of the literature is not homogeneous due to differences in diagnostic criteria, clinical settings, and patient populations. Most of the available studies are case reports or small observational series; thus, while these are useful to describe unusual presentations, they do not allow firm or generalizable conclusions. Future research should make use of multicenter registries or large retrospective cohorts, especially in primary care settings, to build and test predictive models that support clinical decisions and help identify patients who may need earlier referral for suspected serious disease. Although based mainly on case reports, some RF clusters described in the literature [60,82,90] may provide preliminary guidance, but their value needs to be confirmed in studies with experimental designs.

### 4.3. Strengths and Limitations

This review has several strengths. First, the analysis was conducted using a systematic and transparent method, based on the JBI approach [18] and PRISMA-ScR criteria [19].

In addition, the inclusion of heterogeneous sources such as case-based studies and narrative reviews made it possible to identify less frequent clinical presentations and to map a broader overview of the literature. However, some limitations should be acknowledged. The quality of the included studies was not formally assessed. Most consisted of case-based studies or narrative reviews, which provide valuable descriptive information but are methodologically limited and prone to bias. Furthermore, the inclusion of only English or Italian language studies, together with the restriction to publications from 1999 to 2025, may have led to the exclusion of relevant evidence. The variability among the studies and the lack of precision in the description of RFs also made it difficult to group findings and interpret the data consistently. Finally, results from systematic reviews were not directly used to avoid methodological errors related to data duplication, although these reviews were consulted to identify relevant primary studies.

## 5. Conclusions

This scoping review aimed to map the current evidence on the presence and diagnostic utility of RFs for detecting spinal malignancies in patients presenting with low back pain. Although a substantial number of studies were identified, the majority consisted of case-based literature, reflecting a limited availability of high-quality primary research. While certain RFs, such as a history of cancer, showed greater consistency across studies, most others demonstrated poor standalone diagnostic performance. Furthermore, considerable variability was observed in how RFs were defined, applied, and reported. Overall, and in patients with LBP associated with underlying malignancy, RFs seem to be more useful for ruling in rather than ruling out (i.e., screening) serious pathologies. Nevertheless, the findings point to the need for clearer research methods and better-designed studies to help clinicians recognize serious conditions earlier in patients with low back pain. Although current evidence remains limited, assessing RFs in combination rather than in isolation may lead to better diagnostic accuracy. Greater clarity on how RFs are defined, along with the development of reliable clinical tools, could improve clinical decision-making and help detect cancer earlier in patients with low back pain.

## Figures and Tables

**Table 1:** Features of case-based studies.

Variables	Number of Studies
**TOTAL STUDIES**	**30**
***Nation***	
Usa	9
Brazil	7
Portugal	4
Italy	3
Hong Kong	2
Pakistan	2
South Korea	1
Singapore	1
India	1
Denmark	1
Colombia	1
Cile	1
***Year***	
Last 10 years (2014–2025)	22
Previous studies (1999–2013)	8
**Variables**	**Number of Participants**
**TOTAL PARTECIPANTS**	**32**
Men	15
Women	17
***Age***	
≤20 years	5
>20 and ≤ 50 years old	10
>50 and < 65 years old	9
≥65 years old	8
***Setting***	
Direct access to the clinic or hospital	23
Chiropractor clinic/private practice	6
Private practice physiotherapist	3
***Onset***	
Acute spontaneous (until 3 months)	10
Chronic spontaneous (3 months onwards)	18
With causes	3
Not reported	1
***Comorbidities***	
Cancer	14
Smoking and alcohol	2
Chronic exposure wood smoke	1
Cardiac/vascular disorders	4
Neurological Diseases	1
Diabetes	3
Kidney disorders	1
Thyroid Diseases	1
Gastrointestinal Disorders	3
Orthopedic Pathologies	2
Obesity	2
Rheumatic Diseases	1
Gynecological pathologies	2
Pregnancy	1
Not reported	16
***Clinical signs***	
Lumbar pain	32
Abdominal pain	6
Abdominal stiffness	3
Chest pain	1
Fever/Chills	1
Lumbar tenderness	7
Reduced range of movement (ROM)	4
Radiating pain	14
Neurological signs	22
Muscle weakness	9
Reduction in osteotendinous reflexes	3
Presence of palpable mass	2
Scoliosis	2
Dysuria	1
Clenched Fist Percussion Test	1
Supine Sign	1
Renal Murphy sign	1
Asthenia	1
Adynamia	1
Hemoptysis	1
***Images and findings***	
Colonoscopy	2
Cystoscopy	2
Esophagogastroduodenoscopy	1
Fluorescent in situ hybridization	1
Magnetic resonance imaging	23
Scintigraphy	3
Ultrasounds	4
PET positron emission tomography	5
CT Computed Axial Tomography	18
Mammography	1
X-rays	23
Biopsy	20
PSA Testing	2
Blood test	17
***Diagnosis***	
Breast cancer	1
Stomach cancer	2
Lung cancer	2
Colon cancer	2
Prostate cancer	2
Pancreatic cancer	1
Kidney cancer	1
Pelvic cancer	1
Penis cancer	1
Urothelial cancer	1
Appendix cancer	1
Ewing Sarcoma	3
Lumbar Schwannoma	2
Leukemia	1
Primary Bone Liposarcoma	1
Osteoid osteoma	2
Secondary metastatic lesion	17
Cavernous Angioma	1
Giant cell cancer	1
Mastocytosis	1
Neurofibroma	1
Inflammatory myofibroblastic cancer	1
***Reported results***	
Treated	3
Partially care for	9
Dead	5
In treatment	10
Not reported	5

**Table 2:** Red Flags related to case reports, case series, case studies.

Red Flags	Explicitly Reported as Absent	Inferred from Clinical Data	Reported by Authors	Not Mentioned
Failure of conservative treatment	0	17	4	11
Pain duration > 3 months	0	17	0	15
Age > 50	0	16	1	15
Progressively worsening pain	0	11	1	20
Abnormal blood tests	0	7	0	25
Radiating pain	4	9	0	19
Pain duration > 1 month	0	7	0	25
Age < 18	0	4	0	28
Systemic symptoms (fever, chills, night sweats, fatigue, malaise)	9	11	1	11
Night pain	0	6	5	21
Neurological signs (reduced sensitivity, loss of sphincter control, gait deficit, altered reflexes)	9	22	0	5
History of cancer (personal)	2	10	3	17
History of surgery	0	5	0	27
Abdominal pain	3	6	0	23
Motor weakness	1	9	0	22
Unexplained weight loss	3	8	3	18
Family history of cancer	1	5	0	26
Severe and continuous pain	0	4	2	26
C-reactive protein > 10 mg/L	0	3	0	29
Bladder dysfunction (urinary retention or incontinence)	8	11	0	23
Palpable mass	0	1	0	31
Bowel dysfunction	7	11	0	24
Saddle anesthesia	1	2	0	29
Recent trauma	1	1	0	30
Recent infection	0	1	0	31
Smoking history	0	1	0	31
Pregnancy	0	1	0	31
Pain differing from previous episodes	0	1	0	31
Chronic exposure to wood smoke	0	1	0	31
Constant non-provocative pain	0	1	0	31
Improvement with NSAIDs	0	0	2	30
Change in symptom quality and pain resistant to analgesics	0	0	1	31

**Table 3:** Demographic-related RFs.

Figure	Type of Study	Red Flag	Diagnostic Accuracy
Premkumar 2018 [90]	Retrospective observational study	Age > 50	Se 0.717; Sp 0.326; LR+ 1.060 (0.960–1.170); LR− 0.870 (0.680–1.110)
		Age > 70	Se 0.226; Sp 0.795; LR+ 1.100 (0.820–1.470); LR− 0.970 (0.90–1.060)
Henschke 2009 [92]	Prospective Cohort Study	Age > 50	Sp 0.660 (0.630; 0.690)
		Age > 70	Sp 0.950 (0.940; 0.960)

**Table 4:** Medical history related RFs.

First Author and Year of Publication	Type of Study	RF	Diagnostic Accuracy
Tsiang 2019 [93]	Retrospective case–control study	History of cancer	Se 0.917 Sp 0.778
		Night pain	Se 0.542 (0.328; 0.744) Sp 0.496 (0.448; 0.543)
		Pain at rest	Se 0.250 (0.098; 0.467) Sp 0.698 (0.653; 0.740)
		Urinary retention	Se 0.042 (0.001; 0.211) Sp0.958 (0.935; 0.974)
Premkumar 2018 [90]	Retrospective cohort study	History of cancer	Se 0.320; Sp 0.956; LR+ 7.250 (5.650;9.300); LR− 0.710 (0.640; 0.790)
		Night pain	Se 0.554; Sp 0.418; LR+ 0.850 (0.830; 1.100); LR− 1.070 (0.900; 1.270)
		Unexplained weight loss	Se 0.082; Sp 0.956; LR+ 1.870 (1.100; 3.170); LR− 0.960 (0.920; 1.010)
Henschke 2009 [92]	Prospective Cohort Study	History of cancer	Se 0.000 (0.000; 0.000); Sp 0.960 (0.950; 0.970)
		Unexplained weight loss	Se 0.000 (0.000; 0.000); Sp 1.000 (0.990; 1.000)
		Constant, progressive, non-mechanical pain	Se 0.000 (0.000; 0.000); Sp 0.970 (0.960; 0.980)
		Gradual onset before the age of 40	Se 0.000 (0.000; 0.000); Sp 0.910 (0.900; 0.930)
		Insidious Onset	Se 0.000 (0.000, 0.000); Sp 0.830 (0.800; 0.850)
		Systemic malaise	Se 0.000; Sp 0.980 (0.970; 0.980)

**Table 5:** RFs related to physical examination.

First Author and Year of Publication	Type of Study	RF	Diagnostic Accuracy
Henschke 2009 [92]	Prospective Cohort Study	Altered sensation from the trunk down	Se 0.000 (0.000; 0.000); Sp 0.980 (0.970; 0.990)

**Table 6:** Red flag combinations proposed in the literature (based on review data).

First Author and Year of Publication	Type of Study	Red Flag Combinations	Diagnostic Accuracy
Premkumar 2018 [90]	Retrospective cohort study	Combination of unexplained weight loss and history of cancer	Se 0.025; Sp 0.998; LR+ 10.250 (3.600; 29.210); LR− 0.980 (0.950; 1.000)

**Table 7:** Red flag combinations proposed in the literature (based on review data).

First Author and Year of Publication	Type of Study	Red Flag Combinations	Diagnostic Accuracy
Henschke 2013 [60]	Systematic review	Combination of age > 50 years, history of cancer, unexplained weight loss, and failure to improve with conservative therapy	Se 1.000
Delladio 2013 [82]	Narrative review	Age combination > 50 years, history of cancer, unexplained weight loss, no improvement after one month	Se 1.000; Sp 0.600; LR+ 2.400; LR− 0.060;

**Table 8:** Diagnostic accuracy of selected red flags and their combinations for cancer detection in patients with low back pain *.

Red Flag (RF)	Sensitivity	Specificity	LR+	LR−	Source
Age > 50 years	0.717	0.326	1.060	0.870	Premkumar et al. [90]
History of cancer	0.320	0.956	7.250	0.710	Premkumar et al. [90]
Unexplained weight loss	0.082	0.956	1.870	0.960	Premkumar et al. [90]
Constant, progressive, non-mechanical pain	0.000	0.9700	0.000	1.030	Henschke et al. [92]
Combination of unexplained weight loss and history of cancer	0.025	0.998	10.250	0.980	Premkumar et al. [90]
Age combination > 50 years, history of cancer, unexplained weight loss, no improvement after one month	1.000	0.600	2.400	0.060	Delladio et al. [82] (data from secondary sources)
Combination of: history of cancer, age > 50, weight loss, failure of conservative treatment	1.000	—	—	—	Henschke et al. [60] (data from secondary sources)

* Likelihood ratios were either reported in the original studies or calculated from sensitivity and specificity values; all figures are presented with three-decimal precision.

## Data Availability

All data generated or analyzed during this study are included in this published article [and Appendix A, Appendix B and Appendix C].

## Abbreviations

Red flag (RF). Red flags (RFs), Low back pain (LBP), People Concept Context (PCC), Preferred Reporting Items for Systematic Reviews and Meta Analyses extension for Scoping Reviews (PRISMA ScR), Confidence Interval (CI), Likelihood ratio (LR), World Health Organization (WHO), Joanna Briggs Institute (JBI), Sensitivity (Se), Specificity (Sp)

## Appendix A. Search Strategy and Grey Iterature

Date: 15 March 2025		
**DATABASE**	**SEARCH STRATEGY**	**RESULTS**
MEDLINE	((“Low Back Pain”[MeSH Terms] OR “Low Back Pain”[All Fields] OR “low back ache”[All Fields] OR “lumbodynia”[All Fields]) AND (“tumor”[All Fields] OR “cancer”[All Fields] OR “malignancy”[All Fields] OR “neoplasm”[All Fields] OR “tumour”[All Fields]) AND (“red flags”[All Fields] OR “red herrings”[All Fields] OR “red flag”[All Fields] OR “red herring”[All Fields]))	68
WEB OF SCIENCE	(“low back pain” OR lumbago OR “lower back pain” OR “low backache” OR “low back ache”) AND (“differential diagnosis” OR referral OR consultation OR “physical examination” OR inspection OR observation OR screening OR “red flag” OR “red herring” OR “specific pathology” OR “serious pathology” OR “severe pathology” OR “tumor” OR cancer OR “malignancy” OR “neoplasm”)	176
SciELO	(“low NEXT back NEXT pain” OR “lumbar pain” OR “lumbago” OR “low back ache” OR “low backache”) AND (“tumor” OR cancer OR “malignancy” OR “neoplasm” OR “oncological disease” OR “cancer disease”) AND (“differential diagnosis” OR diagnosis OR symptoms OR signs OR findings OR referral OR consultation OR “physical examination” OR inspection OR observation OR screening)	15
COCHRANE LIBRARY	(“low back pain” OR lumbago OR “lower back pain” OR “low backache” OR “low back ache”) AND (“differential diagnosis” OR diagnosis OR symptoms OR signs OR findings OR referral OR consultation OR “physical examination” OR inspection OR observation OR screening) AND (“red flag” OR “red herring” OR “specific pathology” OR “serious pathology” OR “serious disorder” OR “specific disorder” OR “serious disease” OR “specific disease”) AND (“tumor” OR cancer OR “malignancy” OR “neoplasm”)	17
SCOPUS	(“low back pain” OR lumbago OR “lower back pain” OR “low backache” OR “low back ache”) AND (“differential diagnosis” OR diagnosis OR symptoms OR signs OR findings OR referral OR consultation OR “physical examination” OR inspection OR observation OR screening) AND (“red flag” OR “red herring” OR “specific pathology” OR “serious pathology” OR “serious disorder” OR “specific disorder” OR “serious disease” OR “specific disease”) AND (“tumor” OR cancer OR “malignancy” OR “neoplasm”	151
GOOGLE SCHOLAR	“low back pain” AND “differential diagnosis” AND “red flag” AND (“tumor” OR “malignancy”)	195

## Appendix B. Studies Added from Grey Literature

1. Kaur R; Kaur L; Iqbal A; Patel, N. Urothelial Carcinoma With Bone Metastasis Mimicking Sciatica: A Common Neoplasm With an Uncommon Presentation. *Cureus* **2024**, *16*, e55259. https://doi.org/10.7759/cureus.55259
2. Patel, H.G.; Tabassum, S.; Shaikh, S. *E. coli* Sepsis: Red Flag for Colon Carcinoma-A Case Report and Review of the Literature. *Case Rep. Gastrointest. Med*. **2017**, *2017*, 2570524. https://doi.org/10.1155/2017/2570524. PMID: 28695023; PMCID: PMC5485293.
3. Henschke, N.; Maher, C.G.; Ostelo, R.W.; de Vet, H.C.; Macaskill, P.; Irwig, L. Red flags to screen for malignancy in patients with low-back pain. *Cochrane Database Syst. Rev.* **2013**, *2013*, CD008686. https://doi.org/10.1002/14651858.CD008686.pub2. PMID: 23450586; PMCID: PMC10631455.
4. Finucane, L.; Greenhalgh, S.; Selfe, J. What are the Red flags to aid the early detection of metastatic bone disease as a cause of back pain? *Physiother. Pract. Res.* **2017**, *38*, 73–77.
5. Delladio, M.; Maselli, F.; Testa, M. Red flags or red herrings: what is the actual weight of the signs and symptoms of alarm in the evaluation of patients with low back pain/Red flags o red herrings: Qual e il reale peso dei segni e sintomi di allarme nella valutazione del paziente con lombalgia. *Sci. Riabil.* **2013**, *15*, 5+. Available online: https://link.gale.com/apps/doc/A331080126/AONE?u=anon~1cf485a3 (accessed on 9 March 2025).

## Appendix C. Excluded Studies with Reasons

**Author, Year**	**Reference (Title, Doi (or Link If Not Available)**	**Reason for Exclusion**
Menezes, 2025	Clinical and radiological parameters in malignant spinal tumors: A descriptive analysis DOI: 10.1590/1413-785220253301e285913	No specific data on low back pain; only overall numbers of patients with back pain and those with lumbar metastases provided.
Seddio, 2024	The incidence, providers involved, and patient factors associated with diagnosis of specific lumbar spine pathology subsequent an initial nonspecific low back pain diagnosis DOI: 10.1016/j.spinee.2024.10.008	No data on how many patients with LBP developed cancer; no database in Supplementary Materials; no specific RFs reported.
Jenkins, 2024	Diagnostic imaging in the management of older adults with low back pain: analysis from the BAck Complaints in Elders: Chiropractic—Australia cohort study DOI: 10.1186/s12998-024-00562-z	No cancer cases identified.
Lee, 2024	Appropriateness of magnetic resonance imaging of the lumbar spine orders for low back pain in a general hospital DOI: 10.1177/20101058241248208	No information on patients with confirmed cancer diagnosis and associated red flags.
Meidinger, 2023	A Model of Triage of Serious Spinal Pathologies and Therapeutic Options Based on a Delphi Study DOI: 10.3390/medicina59071283	Focuses on hypotheses, not on patients; no cancer analyzed; not relevant
Karpuz, 2023	The Red and Yellow Flag Awareness Level of Family Physicians in Low Back Pain DOI: 10.33880/EJFM.2023120304	No tumor-specific red flags for LBP reported; focused on physiotherapists’ responses; not relevant.
Dixit, 2023	Low Back and Neck Pain DOI: 10.1007/978-3-031-23488-0_46	No full text available
Guerra, 2023	Screen time and low back pain in children and adolescents: a systematic review of Brazilian studies DOI: 10.1590/1984-0462/2023/41/2021342	Focuses on non-specific LBP and telephone use; not relevant to cancer.
Aliaga-Chávez, 2022	Presentación histopatológica atípica en médula ósea de mieloma múltiple DOI: 10.35434/rcmhnaaa.2022.151.1016	Full text with no English or Italian language
Álvarez-Restrepo, 2022	Metanephric Adenoma: differential diagnosis of upper tract urothelial carcinoma. A Case Report DOI: 10.15446/cr.v8n1.92283	No relevant or specified red flags; not relevant.
Melman, 2022	Many people admitted to hospital with a provisional diagnosis of nonserious back pain are subsequently found to have serious pathology as the underlying cause DOI: 10.1007/s10067-022-06054-w	Red flags not extracted; not relevant.
Sporniak, 2022	In search of “red flag” symptoms accompanying spinal pain in diffuse large B-cell lymphoma DOI: 10.20452/pamw.16283	No red flags identified; not relevant.
Konbaz, 2021	Sequestrated Lumbar Disc Herniation Mimicking Spinal Neoplasm DOI: 10.7759/cureus.18529	No patients with cancer reported; not relevant.
Sacoto García, 2021	Evolution of low back pain in cancer patients treated with interventional pain management DOI: 10.20986/resed.2021.3901/2020	Full text with no English or Italian language
Machado, 2020	Emergency department care for low back pain: Should we adopt recommendations from primary care guidelines? DOI: 10.1111/1742-6723.13593	No red flags in patients with LBP and cancer reported; not relevant.
Urrutia, 2020	Management of patients with low back pain in the emergency department: Is it feasible to follow evidence-based recommendations? DOI: 10.1111/1742-6723.13544	Only red flags mentioned, but not associated with specific pathologies; not relevant.
Ceballos, 2020	Malignant tumor of the spine http://scielo.sld.cu/scielo.php?script=sci_arttext&pid=S0864-215X2020000200009&lng=es&nrm=iso Epub 01-Feb-2021 (accessed on 8 March 2025)	Full text with no English or Italian language
Pizarro, 2020	Síndrome de Cushing provocado por carcinoma suprarrenal gigante. Caso clínico DOI: 10.4067/S0034-98872020001101679	Full text with no English or Italian language
Santos, 2020	Antegrade insertion of a double J catheter in the treatment of malignant ureteral obstruction: a retrospective analysis of the results obtained with a modified technique at a university hospital DOI: 10.1590/0100-3984.2019.0090	LBP mentioned in the abstract only as a complication after catheter insertion; not relevant.
Al Somali, 2019	Red Flags” signs among Physician’s with acute back pain In Saudi Arabia DOI: 10.5281/zenodo.2552537	No cancer-related red flags; questionnaire-based study; not relevant.
Carmenathy, 2019	Astrocitoma medular de alto grado no infiltrante. http://scielo.sld.cu/scielo.php?script=sci_arttext&pid=S1028-99332019000500640&lng=es&nrm=iso Epub 29-Oct-2019 (accessed on 8 March 2025)	Full text with no English or Italian language
Cook, 2018	Red flag screening for low back pain: nothing to see here, move along: a narrative review DOI: 10.1136/bjsports-2017-098352	No cancer-specific red flags reported; not relevant.
Strudwick, 2018	Review article: Best practice management of low back pain in the emergency department (part 1 of the musculoskeletal injuries rapid review series) DOI: 10.1111/1742-6723.12907	No specific red flags for LBP with cancer reported; not relevant.
Andrašinová, 2018	Low back pain in the elderly https://www.internimedicina.cz/pdfs/int/2018/03/11.pdf#:~:text=Bolesti%20doln%C3%AD%20%C4%8D%C3%A1sti%20zad%20(low%20back%20pain%20%E2%80%93,pohybuje%20mezi%2013%E2%80%9350%20%%20(Bressler%20et%20al.,%201999) (accessed on 8 March 2025)	Full text with no English or Italian language
Jùnior, 2018	Thoracolumbar epidural arachnoid cyst of difficult clinical management: Case Report. DOI: 10.1590/s1808-185120181701177954	Case report on cysts without tumor signs; not relevant.
López-Ruiz, 2018	Schwannoma pélvico retroperitoneal que simula un leiomioma: reporte de un caso y revisión bibliográfica. DOI: 10.24245/gom.v86i3.1964	Full text with no English or Italian language
Yang, 2017	Low Back Pain in Adolescents: A 1-Year Analysis of Eventual Diagnoses DOI: 10.1097/BPO.0000000000000653	No red flags reported; not relevant.
Bartoloni, 2017	Low Back Pain Imaging Management in the Elderly Population DOI: 10.1007/S40134-017-0194-Z	Red flags not directly mentioned; not relevant.
Cardoso, 2017	Meningioma de localización lumbar en un paciente con virus de inmunodeficiencia humana http://scielo.sld.cu/scielo.php?script=sci_arttext&pid=S2221-24342017000200010&lng=es&nrm=iso (accessed on 8 March 2025)	Full text with no English or Italian language
Garcia, 2017	Return to Work after Breast Cancer http://scielo.isciii.es/scielo.php?script=sci_arttext&pid=S0465-546X2017000100051&lng=es&nrm=iso (accessed on 8 March 2025)	Full text with no English or Italian language
Almeida, 2017	Low back pain—a diagnostic approach DOI: 10.5935/1806-0013.20170034	Cancer not clearly mentioned; not relevant.
Velázquez, 2016	Single cutaneous metastasis due to bladder urothelial carcinoma http://scielo.sld.cu/scielo.php?script=sci_arttext&pid=S1029-30192016000800014&lng=es&nrm=iso (accessed on 8 March 2025)	Full text with no English or Italian language
Braun, 2016	Ivory vertebra: imaging findings in different diagnoses DOI: 10.1590/0100-3984.2014.0103	No red flags reported; focused only on radiologists; not relevant.
Massoud, 2016	Myeloid Sarcoma Presenting as Low Back Pain in the Pediatric Emergency Department DOI: 10.1016/j.jemermed.2016.01.033	Case report included in Verhagen’s systematic review; excluded here to avoid methodological errors; not relevant.
Patel, 2016	ACR Appropriateness Criteria Low Back Pain DOI: 10.1016/j.jacr.2016.06.008	Based on 1996 guidelines (inclusion criteria set from 1999 onwards) and does not distinguish between tumors and infections; not relevant
Goldschmidt, 2016	Presenting Signs of Multiple Myeloma and the Effect of Diagnostic Delay on the Prognosis DOI: 10.3122/jabfm.2016.06.150393	Back pain considered as a generic red flag/symptom; unclear whether patients with back pain had other red flags; classified under ‘no cancer analyzed’; not relevant.
Betancourt, 2015	Generalidades de los tumores de la región sacrococcígea http://scielo.sld.cu/scielo.php?script=sci_arttext&pid=S1608-89212015000300026&lng=es&nrm=iso (accessed on 8 March 2025)	Full text with no English or Italian language
Romo, 2015	Spinal cord compression due to nonleukemic granulocytic sarcoma DOI: 10.1016/j.rccan.2015.03.003	Full text with no English or Italian language
Guzmàn, 2015	Association between diastematomyelia and medullo epithelioma. Case report and literature review DOI: 10.4067/S0717-92272015000200004.	Full text with no English or Italian language
Mabry, 2014	Metastatic cancer mimicking mechanical low back pain: a case report DOI: 10.1179/2042618613Y.0000000056	Case report included in Verhagen’s systematic review; excluded here to avoid methodological errors; not relevant.
Thiruganasambandamoorthy, 2014	Risk factors for serious underlying pathology in adult emergency department nontraumatic low back pain patients DOI: 10.1016/j.jemermed.2013.08.140	No red flags specifically associated with cancer reported; not relevant
Neves, 2014	A formação de profissionais de saúde para a prevenção de lesões musculoesqueléticas ligadas ao trabalho a nível da coluna lombar: uma revisão sistemática DOI: 10.1016/j.rpsp.2014.01.001	Full text with no English or Italian language
Vera Vicuña, 2014	Análisis retrospectivo sobre la utilidad de las herramientas de valoración funcional, en las dolencias lumbares a nivel del ámbito laboral http://scielo.isciii.es/scielo.php?script=sci_arttext&pid=S0465-546X2014000500035&lng=es&nrm=iso (accessed on 8 March 2025)	Full text with no English or Italian language
Finucane, 2013	Metastatic disease masquerading as mechanical low back pain; atypical symptoms which may raise suspicion DOI: 10.1016/j.math.2013.02.009	Included in Verhagen’s systematic review; excluded here to avoid methodological errors; not relevant.
Underwood, 2013	Red flags for back pain DOI: 10.1136/bmj.f7432	Only comments on reviews already included; no cancer-specific red flags reported; not relevant.
Pinho; 2012	Solid variant of aneurysmal bone cist on the distal extremity of the radius in a child DOI: 10.1016/j.rbo.2015.05.005	Full text with no English or Italian language
Calvo-Muñoz, 2012	Prevalence of Low Back Pain during Childoohd and Adolescence. A Systematic Review http://scielo.isciii.es/scielo.php?script=sci_arttext&pid=S1135-57272012000400003&lng=es&nrm=iso (accessed on 8 March 2025)	Full text with no English or Italian language
Martínez Suárez, 2012	Consideraciones generales del dolor lumbar agudo. http://scielo.sld.cu/scielo.php?script=sci_arttext&pid=S1726-67182012000100005&lng=es&nrm=iso (accessed on 8 March 2025)	Full text with no English or Italian language
Boissonnault, 2012	Physical therapists referring patients to physicians: A review of case reports and series DOI: 10.2519/JOSPT.2012.3890	No red flags for cancer-related low back pain reported; not relevant.
Karumanchery, 2012	An unusual case of back pain: A large Pheochromocytoma in an 85 year old woman. DOI: 10.1016/j.ijscr.2011.10.006	No red flags reported; not relevant.
Şahin Onat, 2011	Schwannoma which has only sign mechanical backache: A case report https://www.scopus.com/inward/record.uri?eid=2-s2.0-84864143302&partnerID=40&md5=68066901a498fb5110e86d87c1b82288 (accessed on 8 March 2025)	No full text avaliable
Bálint, 2011	The modern international principles of diagnosing and treating low back pain https://www.scopus.com/pages/publications/79958810472?inward (accessed on 8 March 2025)	No full text avaliable
Oliveira, 2011	Profile of the Population Cared for in a Referral Emergency Unit. DOI: 10.1590/S0104-11692011000300014	Non-specific; LBP only mentioned; not relevant
Fialho, 2011	Musculoskeletal system assessment in an emergency room DOI: 10.1590/S0482-50042011000300005	No mention of cancer-related specific LBP and no database available; not relevant.
Nogueira, 2011	Corpectomia da coluna toracolombar com colocação de cage por acesso único via posterior: técnica cirúrgica e resultados de seis pacientes DOI: 10.1590/S1808-18512011000200003	Full text with no English or Italian language
Maraschin, 2010	Dor lombar crônica e dor nos membros inferiores em idosas: etiologia em revisão DOI: 10.1590/S0103-51502010000400013	Full text with no English or Italian language
Morales, 2010	Plasmocitoma Óseo Solitario X1 DOI: 10.4321/s1699-695x2010000300014	Full text with no English or Italian language
Valle Calvet, 2010	Red flags of low back pain DOI: 10.1016/j.semreu.2009.09.006	Full text with no English or Italian language
González, 2008	Hipernefroma gigante: a propósito de un caso http://scielo.sld.cu/scielo.php?script=sci_arttext&pid=S0034-74932008000100012&lng=es&nrm=iso (accessed on 8 March 2025)	Full text with no English or Italian language
Meneses, 2008	Primary filum terminale ependymoma: a series of 16 cases DOI: 10.1590/S0004-282X2008000400017	No red flags reported; not relevant.
Rectenwald, 2008	A case study of back pain and renal cell carcinoma. DOI: 10.1016/j.jcme.2008.01.001	Included in Verhagen’s systematic review; excluded here to avoid methodological errors; not relevant.
Ángel, 2007	Diagnóstico situacional de las internas del reclusorio de mujeres de Manizales https://revistasojs.ucaldas.edu.co/index.php/hacialapromociondelasalud/article/view/1951 (accessed on 8 March 2025)	Full text with no English or Italian language
Duràn, 2007	Dolor lumbar: enfoque basado en la evidencia. http://hdl.handle.net/10495/18924 (accessed on 8 March 2025)	Full text with no English or Italian language
Gonzalez, 2007	Tumor de la glándula suprarrenal http://ve.scielo.org/scielo.php?script=sci_arttext&pid=S0798-05822007000100010&lng=es&nrm=iso (accessed on 8 March 2025)	Full text with no English or Italian language
Sizer, 2007	Medical Screening for Red Flags in the Diagnosis and Management of Musculoskeletal Spine Pain DOI: 10.1111/j.1533-2500.2007.00112.x	No specific red flags for cancer-related low back pain reported; not relevant
Leerar, 2017	Documentation of red flags by physical therapists for patients with low back pain. DOI: 10.1179/106698107791090105	No mention of low back pain associated with cancer; not relevant.
Rives, 2004	Evaluation and treatment of low back pain in family practice DOI: 10.3122/jabfm.17.suppl_1.s23	No specific red flags for LBP and cancer; only mentioned with infections; not relevant.
Barsa, 2003	Red Flags in the diagnosis and treatment of the low back pain https://www.scopus.com/pages/publications/0141493730?inward (accessed on 8 March 2025)	Full text with no English or Italian language
Hudson, 2002	Low back pain: A simple protocol for GPs https://hdl.handle.net/10520/AJA02599333_3131 (accessed on 8 March 2025)	No tumor-specific red flags reported; not relevant.
Arce, 2001	Recognizing spinal cord emergencies https://www.aafp.org/pubs/afp/issues/2001/0815/p631.pdf (accessed on 8 March 2025)	Insufficient data provided and main cancer-associated red flags not reported; not relevant.

## References

[1] Burton A.K., COST B13 Working Group (2004). European Guidelines for Prevention in Low Back Pain.

[2] Feller D., Giudice A., Maritati G., Maselli F., Rossettini G., Meroni R., Lullo G., Hutting N., Mourad F. (2023). Physiotherapy Screening for Referral of a Patient with Peripheral Arterial Disease Masquerading as Sciatica: A Case Report. Healthcare.

[3] Maselli F., Storari L., Barbari V., Colombi A., Turolla A., Gianola S., Rossettini G., Testa M. (2020). Prevalence and incidence of low back pain among runners: A systematic review. BMC Musculoskelet. Disord..

[4] Maselli F., Esculier J.F., Storari L., Mourad F., Rossettini G., Barbari V., Pennella D., Cataldi F., Viceconti A., Geri T. (2021). Low back pain among Italian runners: A cross-sectional survey. Phys. Ther. Sport.

[5] Maselli F., Testa M. (2019). Superficial peroneal nerve schwannoma presenting as lumbar radicular syndrome in a non-competitive runner. J. Back Musculoskelet. Rehabil..

[6] Blyth F.M., Briggs A.M., Schneider C.H., Hoy D.G., March L.M. (2019). The Global Burden of Musculoskeletal Pain—Where to From Here?. Am. J. Public Health.

[7] GBD 2017 Disease and Injury Incidence and Prevalence Collaborators (2017). Global, regional, and national incidence, prevalence, and years lived with disability for 354 diseases and injuries for 195 countries and territories, 1990–2017: A systematic analysis for the Global Burden of Disease Study 2017. Lancet.

[8] Han C.S., Hancock M.J., Downie A., Jarvik J.G., Koes B.W., Machado G.C., Verhagen A.P., Williams C.M., Maher C.G. (2022). Red flags to screen for vertebral fracture in patients presenting with low back pain. Cochrane Database Syst. Rev..

[9] Hartvigsen J., Hancock M.J., Kongsted A., Louw Q., Ferreira M.L., Genevay S., Hoy D., Karppinen J., Pransky G., Sieper J. (2018). What low back pain is and why we need to pay attention. Lancet.

[10] Galliker G., Scherer D.E., Trippolini M.A., Rasmussen-Barr E., LoMartire R., Wertli M.M. (2020). Low Back Pain in the Emergency Department: Prevalence of Serious Spinal Pathologies and Diagnostic Accuracy of Red Flags. Am. J. Med..

[11] World Health Organization Cancer. https://www.who.int/news-room/fact-sheets/detail/cancer.

[12] Storari L., Piai J., Zitti M., Raffaele G., Fiorentino F., Paciotti R., Garzonio F., Ganassin G., Dunning J., Rossettini G. (2025). Standardized Definition of Red Flags in Musculoskeletal Care: A Comprehensive Review of Clinical Practice Guidelines. Medicina.

[13] National Institute for Health and Care Excellence Suspected Cancer: Recognition and Referral. NICE Guideline NG12. https://www.nice.org.uk/guidance/ng12.

[14] DePalma M.G. (2020). Red flags of low back pain. J. Am. Acad. Physician Assist..

[15] Casazza B.A. (2012). Diagnosis and Treatment of Acute Low Back Pain. Am. Fam. Physician.

[16] Finucane L.M., Downie A., Mercer C., Greenhalgh S.M., Boissonnault W.G., Pool-Goudzwaard A.L., Beneciuk J.M., Leech R.L., Selfe J. (2020). International Framework for Red Flags for Potential Serious Spinal Pathologies. J. Orthop. Sports Phys. Ther..

[17] Maselli F., Palladino M., Barbari V., Storari L., Rossettini G., Testa M. (2022). The diagnostic value of Red Flags in thoracolumbar pain: A systematic review. Disabil. Rehabil..

[18] Joanna Briggs Institute (2020). JBI Reviewer’s Manual. https://jbi.global/.

[19] Tricco A.C., Lillie E., Zarin W., O’Brien K.K., Colquhoun H., Levac D., Moher D., Peters M.D.J., Horsley T., Weeks L. (2018). PRISMA Extension for Scoping Reviews (PRISMA-ScR): Checklist and Explanation. Ann. Intern. Med..

[20] Notarangelo G., Giovannico G., Bruno F., Milella C., Mourad F., Maselli F. (2024). Red flags useful to screen for suspect cancer in patients with low back pain: A scoping review protocol. medRxiv.

[21] Ouzzani M., Hammady H., Fedorowicz Z., Elmagarmid A. (2016). Rayyan-a web and mobile app for systematic reviews. Syst. Rev..

[22] Moher D., Liberati A., Tetzlaff J., Altman D.G., The PRISMA Group (2009). Preferred reporting items for systematic reviews and meta-analyses: The PRISMA statement. PLoS Med..

[23] Grant M.J., Booth A. (2009). A typology of reviews: An analysis of 14 review types and associated methodologies. Health Inf. Libr. J..

[24] Mabry L.M., Ross M.D., Tonarelli J.M. (2014). Metastatic cancer mimicking mechanical low back pain: A case report. J. Man. Manip. Ther..

[25] Finucane L. (2013). Metastatic disease masquerading as mechanical low back pain; atypical symptoms which may raise suspicion. Man. Ther..

[26] Massoud M., Del Bufalo F., Caterina Musolino A.M., Schingo P.M., Gaspari S., Pisani M., Orazi C., Reale A., Raucci U. (2016). Myeloid Sarcoma Presenting as Low Back Pain in the Pediatric Emergency Department. J. Emerg. Med..

[27] Rectenwald R. (2008). A case study of back pain and renal cell carcinoma. J. Chiropr. Med..

[28] Verhagen A.P., Downie A., Maher C.G., Koes B.W. (2017). Most red flags for malignancy in low back pain guidelines lack empirical support: A systematic review. Pain.

[29] Kahn E.A. (2017). A Young Female Athlete with Acute Low Back Pain Caused by Stage IV Breast Cancer. J. Chiropr. Med..

[30] Cataldi F., Brindisino F., Angilecchia D., Andreani A., Giovannico G. (2023). Neoplastic malignant cord compression mimicking low back pain: A case report. Physiother. Res. Int..

[31] Ng G.S.N., Chow I.S.W. (2023). Colorectal Cancer Presenting as Sacral Pain at a Chiropractic Clinic. Cureus.

[32] Bang J.S., Adsul N., Lim J.H., Jang I.T. (2018). Extra-Osseous Ewing Sarcoma of Sciatic Nerve Masquerading as Benign Nerve Sheath Tumor and Presented as Lumbar Radiculopathy: Case Report and Review of Literature. World Neurosurg..

[33] Chu E.C., Trager R.J., Yee W.J., Ng K.K. (2022). Lumbar Schwannoma as a Rare Cause of Radiculopathy in the Chiropractic Office: A Case Report. Cureus.

[34] Brindisino F., Scrimitore A., Pennella D., Bruno F., Pellegrino R., Maselli F., Lena F., Giovannico G. (2022). Aggressive Vertebral Hemangioma and Spinal Cord Compression: A Particular Direct Access Case of Low Back Pain to Be Managed—A Case Report. Int. J. Environ. Res. Public Health.

[35] Leri J.P. (2018). Metastatic Cancer of the Thoracic and Lumbar Spine Presenting as Mid- and Low Back Pain in a Long Distance Runner. J. Chiropr. Med..

[36] Kaur R., Kaur L., Iqbal A., Patel N. (2024). Urothelial Carcinoma with Bone Metastasis Mimicking Sciatica: A Common Neoplasm With an Uncommon Presentation. Cureus.

[37] Patel H.G., Tabassum S., Shaikh S. (2017). *E. coli* Sepsis: Red Flag for Colon Carcinoma—A Case Report and Review of the Literature. Case Rep. Gastrointest. Med..

[38] de Moraes F.B., Cardoso A.L., Tristão N.A., Pimenta W.E., Daher S., de Souza Carneiro S., Barbosa N.P., de Lima Malta N., Ribeiro N.B. (2015). Primary liposarcoma of the lumbar spine: Case report. Rev. Bras. Ortop..

[39] Antunes A.A., Siqueira TMJr Falcão E. (2004). Vesical metastasis of gastric adenocarcinoma. Int. Braz. J. Urol..

[40] Ata F., Yousaf Z., Al Kalaji B.N., Ashour A.A., Fael M., Eun Kim G., Bint IBilal A., Elaiwy O., Jones A. (2022). Back pain as an initial feature of advanced gastric cancer mimicking multiple myeloma: A case report and literature review. Qatar Med. J..

[41] Tan W.J., Lee H.Y., Yap W.M.Q., Nolan C.P., Oh J.Y.-L. (2021). An Atypical Presentation of Metastatic Ewing Sarcoma to the Spine. Indian Spine J..

[42] Mannarini M., Maselli F., Giannotta G., Cioeta M., Giovannico G. (2024). Low back pain as main symptom in Low-grade Appendiceal Mucinous Neoplasm (LAMN): A case report. Physiother. Theory Pract..

[43] Sahu P.K., Shankar Ganesh G. (2020). Physiotherapeutic management of a patient with spinal Schwannoma: A case report. J. Bodyw. Mov. Ther..

[44] Marin J.R. (2007). A Teenage Girl with Acute Back Pain. Clin. Pediatr. Emerg. Med..

[45] Soerensen B. (2011). Mechanical diagnosis and therapy (MDT) approach for assessment and identification of serious pathology. Man. Ther..

[46] Falavigna A., Righesso Neto O., dos Santos J.A., Ferraz F.A. (2004). Cavernous angioma of the cauda equina: Case report. Arq. Neuropsiquiatr..

[47] Arnold P.M., Park M.C., Newell K., Kepes J.J., Thrasher J.B. (2009). Thoracic spinal cord compression secondary to metastatic synovial sarcoma: Case report. Coluna/Columna.

[48] Pitombo P.F., Moura R., Miranda R.H. (2009). Continuous spinal block in a patient undergoing partial gastrectomy:Case report. Braz. J. Anesthesiol..

[49] Pinto-Lopes P., Fonseca F.A., Silva R., von Hafe P., Fonseca E. (2013). Indolent systemic mastocytosis limited to the bone: A case report and review of the literature. Sao Paulo Med. J..

[50] dos Santos M.M., Proença S.M., Reis M.I., Viana R.M., Martins L.M., Colaço J.M., Nunes F.M. (2014). Spontaneous rupture of renal angiomyolipoma during pregnancy. Rev. Bras. Ginecol. Obstet..

[51] Oliveira C., Vital L., Serdoura F., Pinho A.R., Veludo V. (2020). Spondylectomy for primary Ewing lumbar sarcoma in children. Rev. Bras. Ortop..

[52] Ortiz E.R., Duarte A., Leonardo M., Rodríguez V., Jovanny L. (2021). Metástasis pulmonar y pleural en cáncer de pene, una patología infrecuente. Rev. Médica Risaralda.

[53] Relvas-Silva M., Lima E.R., Silva M.R., Neves N. (2024). Laparoscopic-assisted Resection of a Retroperitoneal Lumbar Nerve Root Neurofibroma: A Case Report. Rev. Bras. Ortop..

[54] Pucciarelli L.R.M., Cunha M.C.F.d., Moscardini I.S., Chedid D.A., Paiva M., Miyasaka M., Vieira R.S. (2024). Giant cell tumor with vertebral aneurysmatic bone cyst in a young—A case report. Coluna/Columna.

[55] Madson T.J. (2017). Considerations in physical therapy management of a non-responding patient with low back pain. Physiother. Theory Pract..

[56] Chu E.C., Trager R.J., Lai C.R., Leung B.K. (2022). Presumptive Prostate Cancer Presenting as Low Back Pain in the Chiropractic Office: Two Cases and Literature Review. Cureus.

[57] Ahmad T., Ahmed Hussain M.F., Hameed A.A., Manzar N., Lakdawala R.H. (2014). Conservative surgery for osteoid osteoma of the lumbar vertebrae. Surg. Neurol. Int..

[58] Muñoz Moya J., Alfaro Aguirre M., Leiva Silva M., Kakarieka Weisskopf E., López Sáez M. (2019). Tumor miofibroblástico inflamatorio: Presentación variable de una misma patología. Rev. Chil. Pediatr..

[59] Downie A., Williams C.M., Henschke N., Hancock M.J., Ostelo R.W., de Vet H.C., Macaskill P., Irwig L., van Tulder M.W., Koes B.W. (2013). Red flags to screen for malignancy and fracture in patients with low back pain: Systematic review. BMJ.

[60] Henschke N., Maher C.G., Ostelo R.W., de Vet H.C., Macaskill P., Irwig L. (2013). Red flags to screen for malignancy in patients with low-back pain. Cochrane Database Syst. Rev..

[61] Henschke N., Maher C.G., Refshauge K.M. (2007). Screening for malignancy in low back pain patients: A systematic review. Eur. Spine J..

[62] Janny M., Pasquier M., Descarreaux M., Marchand A.-A. (2023). Diagnosis Value of Patient Evaluation Components Applicable in Primary Care Settings for the Diagnosis of Low Back Pain: A Scoping Review of Systematic Reviews. J. Clin. Med..

[63] Finucane L., Greenhalgh S., Selfe J. (2017). What are the Red flags to aid the early detection of metastatic bone disease as a cause of back pain?. Physiother. Pract. Res..

[64] Krasin E., Schermann H., Snir N., Tudor A., Behrbalk E. (2022). A Quick and Comprehensive Guide to Differential Diagnosis of Neck and Back Pain: A Narrative Review. SN Compr. Clin. Med..

[65] Bardin L.D., King P., Maher C.G. (2017). Diagnostic triage for low back pain: A practical approach for primary care. Med. J. Aust..

[66] Casser H.R., Seddigh S., Rauschmann M. (2016). Acute Lumbar Back Pain: Investigation, Differential Diagnosis, and Treatment. Dtsch. Aerzteblatt Int..

[67] Will J.S., Bury D.C., Miller J.A. (2018). Mechanical Low Back Pain. Am. Fam. Physician.

[68] Kinkade S. (2007). Evaluation and treatment of acute low back pain. Am. Fam. Physician.

[69] Lurie J.D. (2005). What diagnostic tests are useful for low back pain?. Best Pract. Res. Clin. Rheumatol..

[70] Della-Giustina D.A. (1999). Emergency department evaluation and treatment of back pain. Emerg. Med. Clin. N. Am..

[71] Siemionow K., Steinmetz M., Bell G., Ilaslan H., McLain R.F. (2008). Identifying serious causes of back pain: Cancer, infection, fracture. Clevel. Clin. J. Med..

[72] Soar H., Comer C., Wilby M.J., Baranidharan G. (2022). Lumbar radicular pain. BJA Educ..

[73] Becker J.A., Stumbo J.R. (2013). Back pain in adults. Prim. Care Clin. Off. Prac..

[74] Borczuk P. (2013). An evidence-based approach to the evaluation and treatment of low back pain in the emergency department. Emerg. Med. Pract..

[75] Winters M.E., Kluetz P., Zilberstein J. (2006). Back pain emergencies. Med. Clin..

[76] Corwell B.N., Davis N.L. (2020). The emergent evaluation and treatment of neck and back pain. Emerg. Med. Clin. N. Am..

[77] Della-Giustina D. (2013). Acute low back pain: Recognizing the “red flags” in the workup. Consultant 360.

[78] Tavee J.O., Levin K.H. (2017). Low Back Pain. Continuum (Minneap Minn).

[79] Della-Giustina D. (2015). Evaluation and treatment of acute back pain in the emergency department. Emerg. Med. Clin. N. Am..

[80] Hayes K., Huckstadt A., Daggett D. (2006). Acute Low Back Pain in the Emergency Department. Adv. Emerg. Nurs. J..

[81] Harwood M.I., Smith B.J. (2005). Low Back Pain: A Primary Care Approach. Clin. Fam. Pract..

[82] Delladio M., Maselli F., Testa M. (2013). Red flags or red herrings: What is the actual weight of the signs and symptoms of alarm in the evaluation of patients with low back pain/Red flags o red herrings [Qual e il reale peso dei segni e sintomi di allarme nella valutazione del paziente con lombalgia]. Sci. Riabil..

[83] Almeida D.C., Kraychete D.C. (2017). Low back pain—A diagnostic approach. Rev. Dor.

[84] Verhagen A.P., Downie A., Popal N., Maher C., Koes B.W. (2016). Red flags presented in current low back pain guidelines: A review. Eur. Spine J..

[85] Underwood M. (2009). Diagnosing acute nonspecific low back pain: Time to lower the red flags?. Arthritis Rheum..

[86] Reito A., Kyrölä K., Pekkanen L., Paloneva J. (2018). Specific spinal pathologies in adult patients with an acute or subacute atraumatic low back pain in the emergency department. Int. Orthop..

[87] Cooney F., Graham C., Jeffrey S., Hellawell M. (2017). Documentation of spinal red flags during physiotherapy assessment. Br. J. Healthc. Manag..

[88] Chu E.C., Trager R.J. (2022). Prevalence of Serious Pathology Among Adults with Low Back Pain Presenting for Chiropractic Care: A Retrospective Chart Review of Integrated Clinics in Hong Kong. Med Sci Monit..

[89] van Tol F.R., Kamm I.M.L.P., Versteeg A.L., Suijkerbuijk K.P.M., Verkooijen H.M., Oner C., Verlaan J.J. (2023). The use of red flags during the referral chain of patients surgically treated for symptomatic spinal metastases. Neurooncol Pract..

[90] Premkumar A., Godfrey W., Gottschalk M.B., Boden S.D. (2018). Red Flags for Low Back Pain Are Not Always Really Red: A Prospective Evaluation of the Clinical Utility of Commonly Used Screening Questions for Low Back Pain. J. Bone Jt. Surg. Am..

[91] Erausquin T.I., Rosado Pardo J.A., Vital J.M., Sarotto A.J., Besse M. (2024). Manifestación clínica inicial de la lipomatosis epidural lumbar grado III de Naka: Serie de casos. Rev. Asoc. Argent. Ortop. Traumatol..

[92] Henschke N., Maher C.G., Refshauge K.M., Herbert R.D., Cumming R.G., Bleasel J., York J., Das A., McAuley J.H. (2009). Prevalence of and screening for serious spinal pathology in patients presenting to primary care settings with acute low back pain. Arthritis Rheum..

[93] Tsiang J.T., Kinzy T.G., Thompson N., Tanenbaum J.E., Thakore N.L., Khalaf T., Katzan I.L. (2019). Sensitivity and specificity of patient-entered red flags for lower back pain. Spine J..

[94] Feller D., Chiarotto A., Koes B., Maselli F., Mourad F. (2024). Red flags for potential serious pathologies in people with neck pain: A systematic review of clinical practice guidelines. Arch Physiother..

